# Causal explanation of academic enthusiasm based on the interaction of teachers and English language learners: Self-regulation, academic hope, and academic engagement among English language learners

**DOI:** 10.3389/fpsyg.2022.997903

**Published:** 2022-12-12

**Authors:** Siros Izadpanah, Yasaman Mohammad Rezaei

**Affiliations:** Department of English Language Teaching, Zanjan Branch, Islamic Azad University, Zanjan, Iran

**Keywords:** academic engagement, academic enthusiasm, academic hope, interaction, self-regulation

## Abstract

**Introduction:**

The present research investigates the causal explanation of academic enthusiasm based on the interaction between teachers and English learners: self-regulation, academic hope, and academic engagement among English language learners.

**Methods:**

The implementation method of this descriptive research and research design was structural equation correlation. The research’s statistical population included intermediate Zanjan city learners (50,977 people) who were studying in the academic year 2021-2022. This research used multi-stage cluster random sampling to test the proposed model. The following questionnaires were used to collect data: Academic enthusiasm (Fredericks et al.); Teacher-student interaction questionnaire (Moray and Zurich); Academic hope questionnaire (Khormai and Kameri); Self-Regulation Questionnaire (Bouffard) and academic engagement (Zarang). Lisrel software was used for data analysis and evaluation of the proposed model using structural equation modeling.

**Results:**

The results of the present study showed that teacher-student interaction, academic self-regulation, academic engagement, and academic hope are related to students’ academic enthusiasm. The results of the present study also support the role of mediators of academic self-regulation, academic engagement, and academic hope in the causal relationships between teacher-student interaction and academic enthusiasm.

**Discussion:**

Based on this, it can be concluded that with the improvement of teacher-student interaction, the level of academic self-regulation, academic engagement, and academic hope of students increases, and these factors together increase their academic enthusiasm. Therefore, it is suggested that to increase academic engagement, academic self-regulation, and academic hope in the students in the education system, attention should be paid to the position of the students’ academic enthusiasm.

## Introduction

In recent years, the approach of positivist psychology concerning human talents and capabilities has attracted the attention of psychologists. Seligman (2002) has suggested that psychology should not limit itself to the pathological point of view but should pay attention to the positive aspect of human existence and use all the individual and social possibilities to improve the mental health of humans. According to Hammill et al. (2022) point of view and positive psychology, the factors that make a person adapt more and more to the contradictions, needs, challenges, and threats of life are the most fundamental structures investigated by the positive approach. Schools and other educational environments are places where academic challenges, obstacles, and pressures are a constant fact of life (Finn and Rock, 1997; Martin and Marsh, 2006; Patrick et al., 2007).

One of the effective capabilities in education, referred to as strategies for dealing with academic issues and challenges, is academic enthusiasm. Students face various obstacles, challenges, and pressures in their daily academic lives. They face poor grades, threatening self-esteem, and diminished motivation, which provide them with a source of stress (Samadieh and Sadri, 2022). One of the protective factors against these challenges, which plays an important role in helping to solve problems and improve their academic achievement, is academic enthusiasm (Jung and Ryu, 2022).

Self-regulation is one of the scopes in which its application is of great importance and leads to students’ academic success. The theory of self-regulatory learning was proposed by Pintrich and de Groot (1990). Self-regulated learning is also a type of learning in which people personally initiate and direct their efforts instead of relying on teachers, parents, or other educational factors to acquire knowledge and skills. Self-regulation is psychological effort to control one’s inner state, processes, and functions to achieve higher goals. Zimmerman and Schunk (2004) believe that in terms of metacognition, motivation, and behavior, self-regulated learners initiate and direct learning processes themselves.

Another effective factor that plays an important role in students’ academic enthusiasm is academic hope (Wu and He, 2022). Hope is a cognitive capacity based on a mutual feeling arising from the purposeful determination of goals and the path to achieving goals (Chena et al., 2020). The importance of hope for academic activities is such that experts like Snyder et al. (2002) and Pekrun (2000) have proposed a concept called academic hope.

Academic engagement is a concept that was first used to explain academic failure and has been used as a basis for reformist actions in education (Fredricks et al., 2004). Academic engagement is a basic principle in academic achievement, rate, speed, graduation rate, and behaviors with a lower risk factor. The term refers to engagements in the institute environment and extracurricular activities such as sports and music. It also includes aspects of the curriculum and students’ relationships with the teacher and other classmates. Finally, academic engagement in learning activities is related to specific aspects of the activity or structure (Fernandez et al., 2016). Many factors are involved in academic engagement, including the cognitive ability that the phrase is one of the neural processes involved in the acquisition, processing, storage, and use of information (Ma and Wang, 2022; Ng et al., 2022). Cognitive ability is the ability that accompanies the process of physical development and allows individuals to process different information. In other words, it is part of the ability to think, reason, speak, remember, organize, understand, and comprehend (Serrano et al., 2022).

## Review of literature

Academic enthusiasm refers to behaviors that are related to learning and academic achievement (Ibrahim et al., 2022). In theories of learning, motivation and passion are a combination of behavioral and cognitive approaches (Grohman and Snyder, 2022). Academic enthusiasm includes how institutions organize resources, learning opportunities, and services to encourage students to participate in such activities and gain benefits (Mahmodiyan et al., 2018). People with academic enthusiasm pay more attention to the issues and topics they are learning, work hard, enjoy their academic duties, show more commitment to the rules of the academic location, avoid inappropriate and undesirable behaviors and perform better in exams (Jafaripour et al., 2020). Overall, academic enthusiasm can protect students against academic adversity as a supportive factor. Accordingly, it is necessary for researchers in the field of education to identify the factors affecting academic enthusiasm and the process of influencing these factors at the top of their research.

Academic enthusiasm includes how institutions organize their learning opportunities, resources, and services in such a way as to encourage learners to participate in such activities and gain benefits (Rashidi et al., 2021). Concerning the academic enthusiasm of the learners, it should be mentioned that academic enthusiasm refers to the amount of energy that a learner spends to carry out her academic activities, as well as the level of effectiveness and efficiency achieved(Chaya, 2022). In other words, in the definition of the university’s academic enthusiasm, the concepts of participation in social activities—the feeling of belonging and valuing the university, the educational environment for academic enthusiasm, and the consequences that the sufficiency of learning are considered important (Jung and Ryu, 2022). Learners who have academic enthusiasm pay more attention and focus on the issues and topics aimed at learning—they commit more to the rules and regulations of the school, avoid inconsistent and unrestrained behaviors, and have more cooperative behaviors (Maslach et al., 2010). In various models of academic enthusiasm, this concept includes two dimensions: obsessive enthusiasm and harmonious enthusiasm (Huang et al., 2021).

In other models, academic enthusiasm is considered an instrument consisting of behavioral, cognitive, and motivational dimensions (Linnenbrink and Pintrich, 2003). A positive attitude toward academic enthusiasm is effective on psychological variables. Behavioral dimension, academic achievement, cognitive dimension of self-regulation, learning goals, flexibility in problem-solving, preference for hard work, and positive coping with hard work (Zhang et al., 2018). The motivational dimension predicts adaptation and class attendance (Wang et al., 2011). The research results indicate that the general enthusiasm for the university causes a decrease in academic failure and increase academic involvement (Archambault et al., 2009). The results of the study of learning and all dimensions of academic enthusiasm among students showed that there is a positive and significant correlation between the teaching environment. Mahler et al. (2018) also concluded in their structural model that students’ academic involvement level can be predicted according to the variable of academic enthusiasm.

### Self-regulation

Self-regulation means self-motivated thoughts, feelings, and actions designed to achieve personal goals (Zimmerman, 2000). From the perspective of Zimmerman (2000), as one of the cognitive-social theorists, self-regulation learning is a type of learning in which learners, instead of those who rely on teachers, parents, and other educational agents to acquire skills and knowledge, personally initiate and guide their efforts. Bouffard et al. (1995) summarized self-regulatory cognition in three metacognitive components using the Pentrich and motivational model. The metacognitive component includes students’ general plans and plans for learning, such as using strategies for organizing and scheduling studies and summarizing materials. The cognitive component includes students’ strategies to retain, learn, and better understand the material. The motivational component includes the students’ level of interest, persistence, and motivation concerning the course material (Bouffard et al., 1995). Self-regulation processes are done in three steps. (1) Anticipation or before the action (in this stage, thinking and planning to do the work takes place). (2) Performance or during the action and 3-Self-reflection, judgment, evaluation, and reaction to one’s activities are (Zimmerman, 2000). Various research shreds of evidence indicate the influential role of family, peers, and teachers in students’ academic self-regulation (Farley and Kimaspoon, 2017). Also, when students have self-regulation skills, they feel efficient and competent, engage in academic activities with internal motivation, and enjoy better academic performance (Morosanova et al., 2016; Blasiman et al., 2017). Based on this, it seems that self-regulated learners act to use educational opportunities and remove academic obstacles and at the same time monitor their actions (Zimmerman and Schunk, 2004). In this way, these learners work effectively in areas of control: Impulse, time management, and coping with psychological pressure caused by the educational context (Murtagh and Todd, 2004). Also, self-regulation also leads to academic engagement, as a study by Morosanova et al. (2016) showed that students’ self-regulated learning skills, which teachers at a high level evaluated, led to high academic engagement in the classroom. Also, students whose self-regulation and class autonomy increase during the transition period to high school, are less likely to decrease their academic engagement (Morosanova et al., 2016). According to Bandura (1977), self-regulation uses the abilities and capabilities of self-direction, self-control, and autonomy. According to him, these abilities are influenced by people’s beliefs about self-regulation in various activities and behaviors. The results of Teng and Zhang’s (Teng and Zhang, 2022) research showed that students with higher self-regulation also have higher academic achievement and have more motivation and interest to continue their education. He also noted that students with lower self-regulation are likely to have lower levels of metacognitive knowledge.

### Academic hope

Academic hope is a belief or expectation of education, a belief by which one expects to achieve positive educational results (Hansen et al., 2014). The review and study of more than 20 years of research in the field of hope show that students who are more hopeful in education and life show better performance than other students and have higher academic enthusiasm (Farid and Ashrafzade, 2021; Sohrabi et al., 2021; Zhong et al., 2021). The results of Mana et al. (2022) research showed that hope is one of the influencing variables on academic self-regulation, academic enthusiasm, and well-being. Also, Chena et al. (2020) showed in research that there is a positive and significant relationship between hope and academic enthusiasm. The results of Laslo-Roth et al. (2022) also showed that people with higher academic enthusiasm have higher academic motivation, are more hopeful about their academic future, and feel higher self-regulation. A study of more than 20 years of research in the field of hope shows that more hopeful students in education and life, show better performance than other students and have higher academic enthusiasm (Wu and He, 2022).

### Academic engagement

Academic engagement is a structure that was first used to explain the failure of academic achievement and was proposed as a basis for reformist measures in the field of education (Fredricks et al., 2004). Academic engagement has been expressed as a basic principle in academic progress, the rate, and speed of completing education, and behaviors with a lower risk factor (Perkmann et al., 2021). In general, academic engagement refers to an emotional and positive state of mind characterized by characteristics such as high levels of energy-interest and being immersed in activities so that a person does not notice the passage of time (Valadez et al., 2021). Linnenbrink and Pintrich (2003) also proposed three dimensions for academic engagement: Behavioral engagement is the visible behaviors of students in dealing with homework (which includes the components of homework effort, persistence in homework, and asking for help from others; cognitive engagement is a variety of processing processes used by learners for learning and includes the components of using cognitive and metacognitive strategies. Motivational (emotional) involvement is having positive feelings during learning and preventing negative feelings such as anxiety, which includes the components of feeling, value, and emotion.

One of the factors related to the school and participation in the education process, which plays an effective role in the students’ academic enthusiasm and is related to academic self-regulation, academic hope, and academic engagement is the interaction between the teacher and the student(Roorda et al., 2019; Scales et al., 2019). The teacher and the students spend at least a quarter of their time in school, and establishing effective communication improves their feelings toward school and improves the quality of teaching and learning (Pianta et al., 2022). Teacher-student interaction is an important source of social support that has a central plan in students’ learning and is the main aspect of the teaching profession. It contributes to educational outcomes (Lan and Moscardino, 2019). Teacher-student interaction is one of the social and psychological factors of the class, which refers to class management - classroom and non-classroom communication between teachers and students (Zendarski et al., 2020). Pianta et al. (2022) define student-teacher communication as part of positive emotions; self-confidence in mutual communication. Martin and Marsh (2006) have concluded that there is a direct relationship between academic enthusiasm and teacher-student relationship, self-regulation, and academic engagement. In their research, Roorda et al. (2019) concluded a positive and meaningful relationship between self-regulation beliefs and teacher-student interaction. Scales et al. (2019) also concluded in their research that the teacher-student relationship is an indirect predictor of academic enthusiasm. Also, the results of Serrano et al.'s (2022) research showed that students with positive and constructive relationships with teachers have higher academic interests, academic engagement, success, self-regulation, hope, and academic enthusiasm than others. In research, Roorda et al. (2019) showed that the quality of communication between teachers and students plays a key role in students’ academic performance. The high level of teacher support leads to academic interest, engagement, self-regulation, academic enthusiasm, and academic hope in students. The results of Crouch’s research also showed that students who establish supportive and friendly relationships with teachers have positive academic attitudes, satisfaction with school, academic engagement, and more hope to continue their studies (Crouch et al., 2018).

Regarding the necessity of this research, it can be said that the educational environment is one of the environments that require adaptation and adaptability, in fact, the challenges and pressures of the educational environment, on the one hand, and the rapid cognitive and emotional changes on the other hand - the need for learners increases it to have it. In general, academic enthusiasm can protect the learner against academic difficulties as a protective factor. Based on this, it is necessary for researchers in the field of education to identify the factors that affect academic enthusiasm and the process of influencing academics. In this regard, considering the relationship between academic enthusiasm and academic self-regulation - academic hope and student-teacher interaction on the one hand, and the relationship between academic self-regulation, academic hope, and academic enthusiasm, on the other hand, shows the study of causal explanation of academic enthusiasm based on the interaction of teachers and English language learners: self-regulation, academic hope, and academic engagement among English language learners.

### Hypotheses

(H_1_) The interaction between teachers and language learners significantly affects the academic enthusiasm of English language learners.

(H_2_) Self-regulation significantly affects the academic enthusiasm of English language learners.

(H_3_) Academic hope significantly affects the academic enthusiasm of English language learners.

(H_4_) Academic engagement significantly affects the academic enthusiasm of English language learners.

(H_5_) The interaction of teachers and language learners has a significant effect on the academic enthusiasm of English language learners with the mediating role of self-regulation, academic hope, and academic engagement.

## Materials and methods

### Design of study

Considering the nature of the subject and the purpose of the research, the implementation method of this descriptive research and the correlation research design is based on Structural Equation Modeling (SEM). Using this method, the direct and indirect effects of the variables in the assumed model can be investigated. In this research, Lisrel software was used for data analysis.

### Statistical population

The research’s statistical population included intermediate Zanjan city learners (50,977 people) who were studying in the academic year 2021–2022. The total number of language training centers was 24 (14 for girls and 10 for boys). This research used multi-stage cluster random sampling to test the proposed model. In this way, first, among the 24 intermediate-level institutions in Zanjan city (14 girls’ institutions and 10 boys’ institutions, boys’ institutions were selected). Four boys’ institutions were randomly assigned (4 classes were randomly selected from each institution for research and answering questions). In the current study, all intermediate-level language learners, whose number was 412, were investigated by the census method. Of these, 25 people did not want to answer, 26 questionnaires were incomplete, and a total of 361 correct questionnaires were examined. To carry out the research, first, the necessary permission was obtained to conduct the research and distribute the questionnaires in intermediate-level institutions in Zanjan city. In the next stage, questionnaires were distributed among the learners while making the necessary arrangements with Zanjan institutions within 1 month by referring to the institutions and informing managers and officials about the research objectives. The average time to answer the questionnaires was 45 min.

In this research, the following questionnaires were used to collect data:

*Academic enthusiasm (*Fredricks et al., 2004*):* This questionnaire has 15 items and measures it with a five-point Likert scale. This questionnaire has a subscale of behavioral desire with items 1, 2, 3, and 4, emotional desire with items 5, 6, 7, 8, 9, and 10, and cognitive desire with items 11, 12, 13, 14, and 15. In terms of the psychometric properties of the instrument, Fredricks et al. (2004) reported a standard reliability coefficient of 0.86. Various experts in this field have confirmed the validity of the questionnaire. Cronbach’s alpha coefficient was used to determine the internal consistency of the criterion. The coefficient of reliability (Cronbach’s alpha) for the criterion of academic enthusiasm in Mirsafian’s (2019) research was equal to 0.88.

Murray and Zvoch (2011) *Teacher-student interaction questionnaire:* This questionnaire consists of 17 items that have three subscales of communication (8 questions), trust (5 questions), and alienation (4 questions). Questions are graded on a four-point scale (never, sometimes, often, and always) on a scale of 1 to 4, respectively. In Murray and Zvoch (2011), Cronbach’s alpha coefficients of the communication subscales were 0.89, trust was 0.84 and alienation was 0.72. The convergent and divergent validity coefficients of all three subscales of this questionnaire were measured by the Social Support Scale for Children and Adolescents, with the intimacy component in the communication network questionnaire and with the teacher ranking of friendship (closeness)on the teacher-student relationship scale reported significant.

Also, in Mansouri et al. (2015) research, Cronbach’s alpha coefficient for the whole scale and three subscales of communication, trust, and alienation were 0.83, 0.80, 0.75, and 0.75, respectively. The coefficient construct validity using confirmatory factor analysis showed that the materials related to the subscales of the learner-teacher interaction questionnaire had acceptable factor loads greater than 0.30 and had a positive and significant load on the relevant factor. In this study, Cronbach’s alpha test was used to evaluate the reliability of this instrument, which for the components of communication, trust and alienation were 0.85, 0.75, and 0.78, respectively, and the total alpha of the instrument was 0.86.

*Academic hope questionnaire* (Khormaee and Kamari, 2017): This scale consists of 27 items that measure academic hope in four dimensions: hope for opportunities, hope for obtaining life skills, hope for school usefulness, and hope for obtaining qualifications based on the Likert scale that measures five degrees from strongly disagree (1) to strongly agree (5). The validity of this tool has been investigated in Khormaei and Kamaris’ research (2017) using exploratory factor analysis by the principal components method. The results indicate the existence of four subscales in the materials of this scale, which explains 58% of the total variance of the sample.

Also, the validity of this tool in this study by Cronbach’s alpha method for each of the dimensions of hope for obtaining opportunity, hope for obtaining life skills, hope for school usefulness, hope for obtaining a qualification, and the total score of the academic hope scale is equal to 0.76, respectively. 0.80, 0.90, 0.90 and 0.90 have been reported. In Kamari et al. (2015) research, Cronbach’s alpha coefficients of hope for obtaining opportunities were 0.86, life skills 0.84, school usefulness 0.75, qualifications 0.80, and the whole academic achievement scale was 0.90. In general, the results related to Cronbach’s alpha coefficients were the optimal validity of the scale that showed the academic hope scale.

Also, in Barani et al. (2019) research, Cronbach’s alpha coefficient for each of the dimensions of hope for the opportunity, hope for life skills, hope for school usefulness, hope for qualification, and total score of hope scale is equal to 0.74, 0.80, 0.78, 0.79 and 0.87 were obtained. In this study, Cronbach’s alpha test was used to evaluate the reliability of this tool, which for the components of hope for opportunities, hope for life skills, hope for usefulness in school, and hope for qualification, respectively, were obtained 0.76, 0.85, 0.79, and 0.74 and the total alpha value was 0.90.

Bouffard et al. (1995) *Self-regulatory questionnaire*: The 14-item Bouffard et al. (1995) questionnaire is a self-regulatory assessment tool based on Bandura’s social cognitive theory. Questions are on the Likert scale and measure two factors of cognitive and metacognitive strategies (quoted by Kadivar, 2001). The Likert scale’ three experts determined the validity of the academic engagement questionnaire is from strongly agree (score 5) to strongly disagree (score 1) and questions 5-13-14 are scored in reverse. Kadivar (2001) has studied the validity and reliability of this tool. The construct validity of this questionnaire was reported to be optimal by using correlation coefficients and factor analysis of correlation coefficients between the questions of the questionnaire and Cronbach’s alpha coefficient for measuring internal consistency was 0.08. Based on this, it can be said that this questionnaire can predict the actual scores of the subjects.

*Academic engagement (*Zerang, 2012*):* Academic engagement, a questionnaire designed by Zerang (2012) based on theoretical foundations (Theoretical Model of Linen Bering and Paintingg) is used. This scale has three dimensions of cognitive, motivational, and behavioral academic engagement. Its cognitive dimension is measured with 19 items, its motivational dimension with 10 items and its behavioral dimension with 9 items and they make a total of 38 items. In this questionnaire, the options are set based on a 5-point Likert scale (always true = 5, sometimes true = 4, sometimes true and sometimes false = 3, sometimes false = 2 and always false = 1).

Three experts determined the validity of the academic engagement questionnaire in educational sciences by determining the validity of the content through specialized arbitration and in terms of compliance with its theoretical basis. In this way, the advantages of academic engagement were extracted from theoretical foundations, and phrases were set for each component. Then, by performing a preliminary test for the academic engagement questionnaire by Cronbach’s alpha test, the total reliability of the questionnaire in the preliminary stage with 38 questions was 0.92 and the internal consistency of the subscales of cognitive engagement with 0.84, behavioral engagement 0.76 and motivational engagement is 0.86. Also, the reliability of the whole questionnaire in the final stage was obtained with 38 questions of 0.90 and the internal consistency of the subscales of cognitive engagement is 0.83, behavioral engagement is 0.73, motivational engagement is 0.80, which are desirable and acceptable levels.

Therefore, the academic engagement questionnaire and its subscales have a desirable and acceptable internal consistency. The results of confirmatory factor analysis, 3 factors, and 38 items of the academic engagement questionnaire according to Berg and Pentrich model were confirmed and it was found that the components and expressions of this questionnaire have a suitable factor load (Zerang, 2012).

To use structural equations, the validity of the factor is required, the results of which are presented in the table below: 9514535641.

According to Table 1, the KMO values for teacher-student interaction, academic hope, academic engagement, self-regulatory, and academic enthusiasm questionnaires are 0.726, 0.938, 0.923, 0.829, and 0.875, respectively. The data volume is suitable for factor analysis and also according to the amount of surface covered Chi-square statistic (significance level) of Bartlett index for all variables and their dimensions was equal to (0.001) which was less than 0.01 level and showed that the data have a good correlation.

**Table 1:** KMO and Bartlett test to evaluate the adequacy of sampling and data correlation.

Variable	KMO	Bartlett's Test of Sphericity	Df	Sig
Teacher-Student Interaction	0.726	3,158.494	136	0.001
Academic Hope	0.938	9,275.803	351	0.001
Academic Engagement	0.829	12,189.835	171	0.001
Self-Regulatory	0.923	4,261.154	91	0.001
Academic Eagerness	0.875	2,053.294	105	0.001

Cronbach’s alpha coefficient was used to evaluate the reliability of the research questionnaires and the results showed that Cronbach’s alpha for teacher-student interaction was 0.789, academic hope was 0.968, academic engagement was 0.955, self-regulatory was 934, and academic eagerness equal to 0.872 and above 0.7 that shows the reliability of the questionnaires are appropriate.

## Results

To analyze the analysis data in two sections of descriptive statistics and inferential statistics, SPSS 25 and AMOS 24 software are used. First, the descriptive statistics section shows the central indicators and the research variables’ dispersion. The results are presented in Table 1.

The results of descriptive statistics in Table 2 show the mean of teacher-student interaction variables, academic hope, academic engagement, self-regulatory and academic enthusiasm are 46.990, 81.461, 50.906, 41.787, and 42.029, respectively, with deviations of 8.418, 24.029, 18.086, 12.941 and 10.187. Also, the Skewness and Kurtosis indices are in the range (2-, 2) and show that the distribution of variables is almost normal.

**Table 2:** Central indicators and dispersion of research variables.

Variable	*N*	Mean	Std. deviation	Minimum	Maximum	Skewness	Kurtosis
Teacher-Student Interaction	361	46.990	8.418	25	78	−0.250	0.269
Academic Hope	361	81.461	24.029	40	122	−0.182	−1.311
Academic Engagement	361	50.906	18.086	23	89	0.266	−1.158
Self-Regulatory	361	41.787	12.941	18	67	−0.205	−1.123
Academic Eagerness	361	42.029	10.187	20	68	0.095	−0.456

### Assumptions of using structural equation-path analysis method

#### Default 1: Kolmogorov–Smirnov test

To evaluate the normality of the research variables, the Kolmogorov–Smirnov normality test is used, the results of which are as follows:

Table 3 shows that the sig value is greater than 0.05 and the assumption of normal data is accepted.

**Table 3:** Evaluation of normality of variables by Kolmogorov–Smirnov test.

Variable	Test Statistic (K-S)	Result	Sig
Teacher-Student Interaction	0.022	The distribution of the variable is normal	0.200
Academic Hope	0.025	The distribution of the variable is normal	0.200
Academic Engagement	0.041	The distribution of the variable is normal	0.096
Self-Regulatory	0.035	The distribution of the variable is normal	0.136
Academic Eagerness	0.045	The distribution of the variable is normal	0.081

#### Default 2: Pearson correlation test

To investigate the correlation of research variables, the Pearson test was used, the results of which are as follows:

The results of the Pearson test in Table 4 showed:

**Table 4:** Correlation between research variables.

	1	2	3	4	5
1-Teacher-student interaction	-				
2-Academic hope	0.709^**^	-			
3-Academic engagement	0.676^**^	0.688^**^	-		
4-Self-regulatory	0.692^**^	0.683^**^	0.630^**^	-	
5-Academic eagerness	0.605^**^	0.572^**^	0.653^**^	0.589^**^	-

**Correlation is significant at the 0.01 level (2-tailed).

1. The correlation coefficient of academic enthusiasm with teacher-student interaction is equal to 0.605, with academic hope equal to 0.572, with academic engagement equal to 0.653, and with self-regulatory equal to 0.589 and at the level of 0.01 is significant.

2. The correlation coefficient of self-regulatory with teacher-student interaction is 0.692, with academic hope is 0.683, with academic engagement is 0.630 and at the level of 0.01 is significant.

3. The correlation coefficient of academic engagement with teacher-student interaction is equal to 0.676, with academic hope is equal to 0.688 and at the level of 0.01 is significant.

4. The correlation coefficient of academic hope with teacher-student interaction is equal to 0.709 and is significant at the level of 0.01.

**Figure 1:**
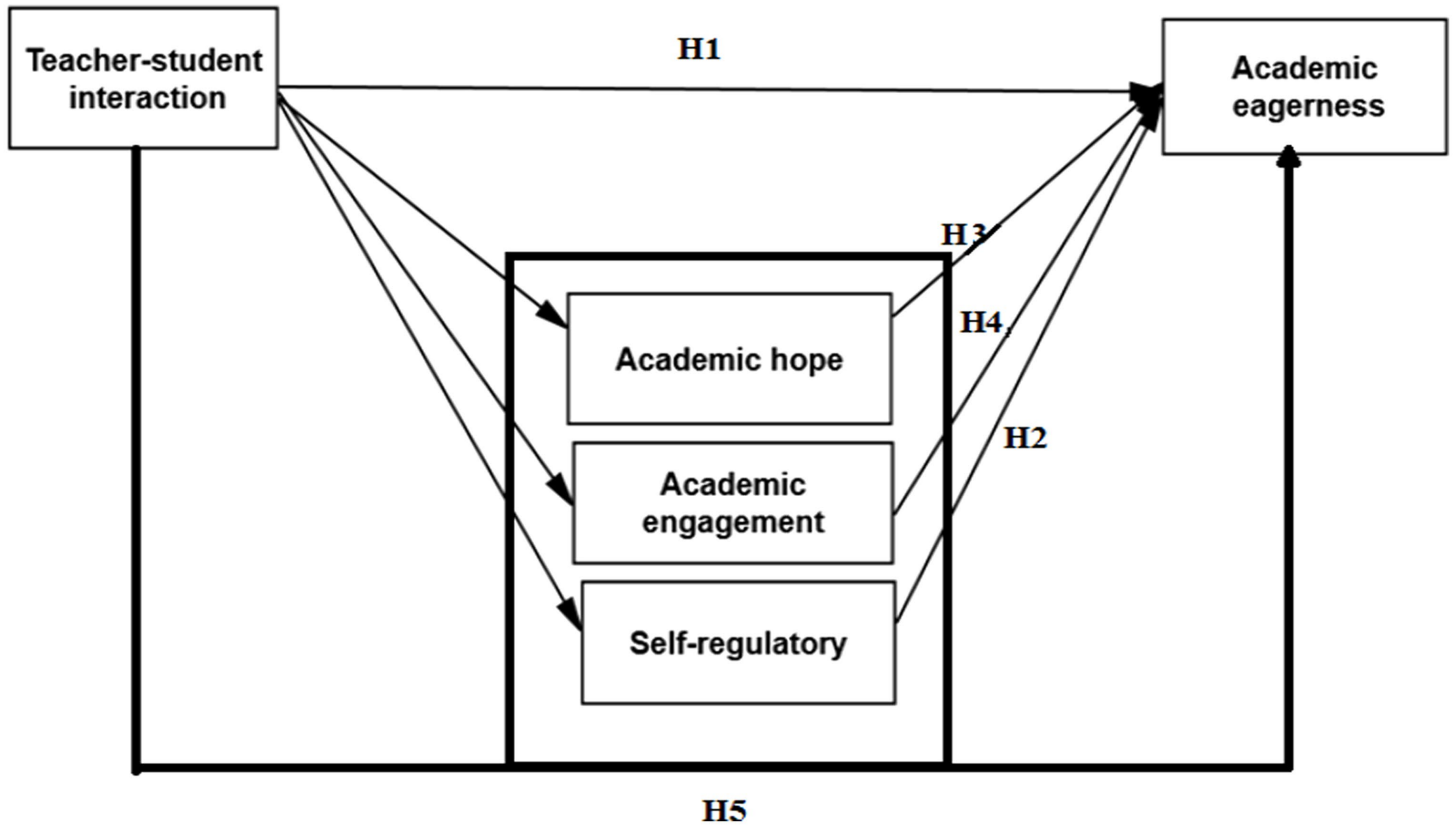
Theoretical framework figure.

**Figure 2:**
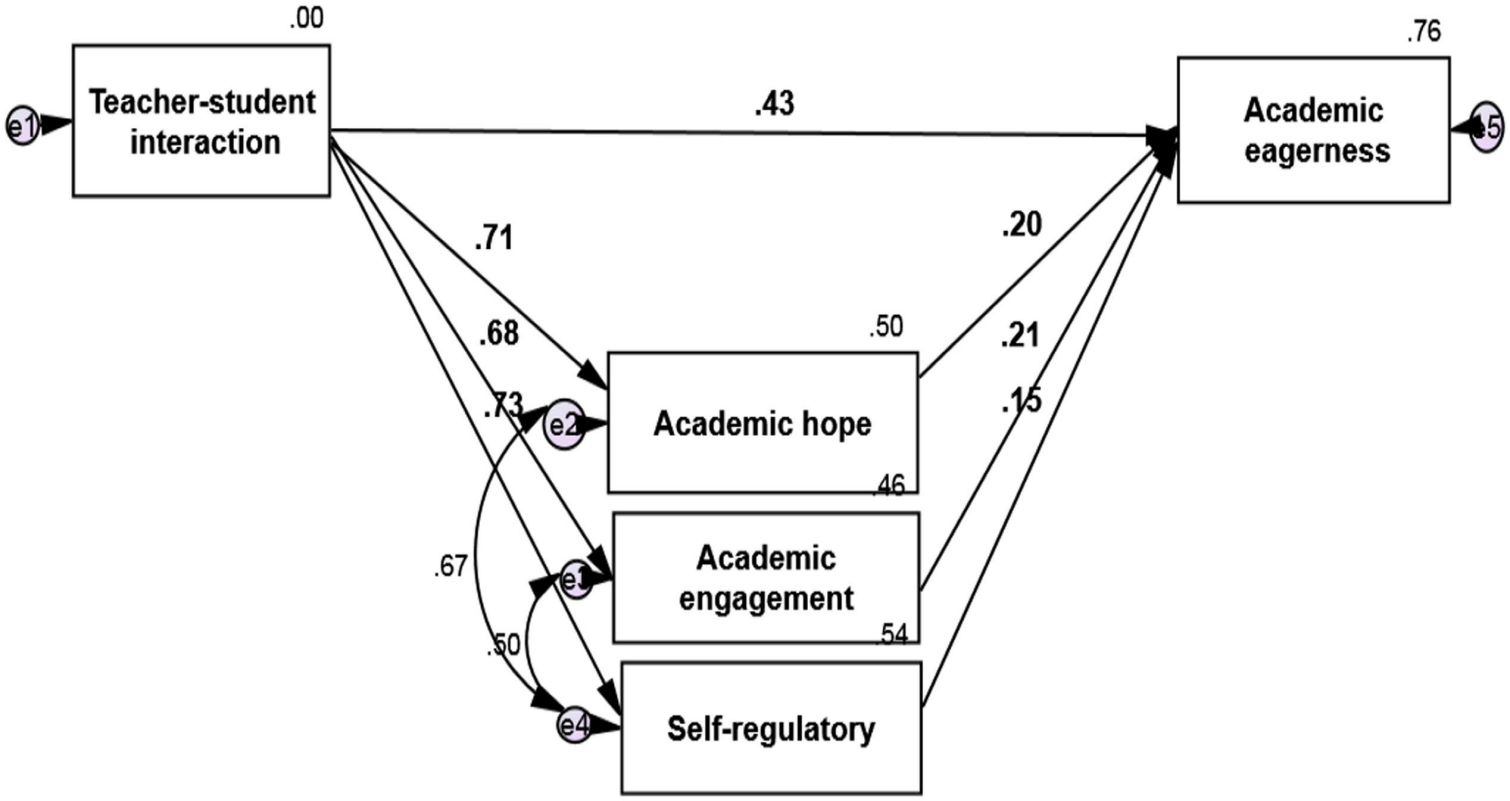
Model fit in standard estimation mode.

**Figure 3:**
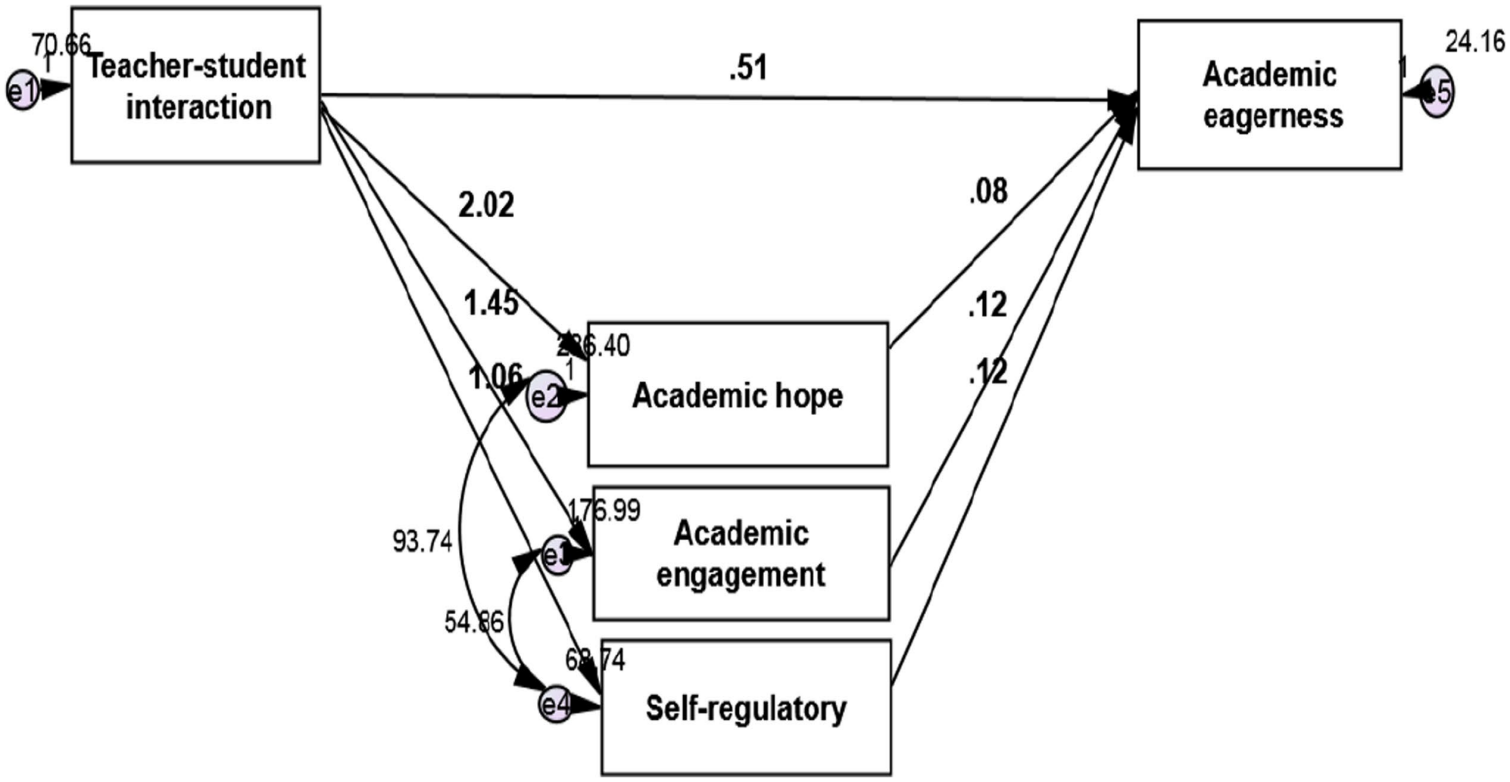
Model fit in non-standard estimation mode.

The path analysis method with Amos 24 software is used to investigate the relationship between research variables. The research model is as (Figures 1-3):

According to the software output, the calculated value of *χ*^2^ equals 2.989, which is less than 3 concerning its degree of freedom. The low value of this index indicates a slight difference between the conceptual model and the observed research data. The RMSEA value equals 0.074 and is less than the critical point of 0.08. Goodness-of-fit index (GFI), Normed Fit Index (NFI), Incremental Fit Index (IFI), Relative Fit Index (RFI), and Comparative Fit Index (CFI) indices are equal to 0.940, 0.965, 0.966, 0.893, and 0.965, respectively, and is greater than the critical point of 0.9, which indicates a high fit. In the following, the research hypotheses are examined.

The results of the relationship analysis are presented in Table 5:

**Table 5:** Relationships between research variables.

Hypotheses		Path coefficients in standard estimation mode	*T*-value	*p*-value	Status
H_1_	The interaction between the teacher and the students → the academic enthusiasm of the English language learners	0.428	10.810	0.001	Confirmation
H_2_	Self-regulation → academic enthusiasm of English language learners	0.151	2.199	0.028	Confirmation
H_3_	Academic hope → Academic enthusiasm of English language learners	0.197	3.289	0.001	Confirmation
H_4_	Academic engagement → academic enthusiasm of English language learners	0.208	4.186	0.001	Confirmation
H_5_	Interaction between teacher and students → self-regulation, academic hope, and academic engagement → academic enthusiasm	0.455	-	0.013	Confirmation

*H1*: Teacher-student interaction significantly affects English students’ academic enthusiasm.

The results of path analysis in Figure 2 and Table 5 show that the standard coefficient between teacher-student interaction and English students’ academic enthusiasm is 0.428. According to the absolute value of t-test statistics which is equal to 10.810 and greater than 1.96, it can be concluded with a 99% probability of teacher-student interaction with a positive effect and is significant on the academic enthusiasm of English language learners (value of *p* = 0.001; *β* = 0.428) In other words, in exchange for increasing one unit of teacher-language interaction, the academic enthusiasm of English language learners increases by 0.428 units.

*H2*: Self-regulation significantly affects English students’ academic enthusiasm. The results of this hypothesis are presented in Table 5.

The results of path analysis in Figure 2 and Table 5 show that the standard coefficient between self-regulation and academic engagement of English language learners is 0.151. Consistent with the absolute value of t-test statistics which is equal to 2.199 and greater than 1.96, it can be concluded, with a 95% probability, that self-regulation has a positive and significant effect on the academic enthusiasm of language learners (value of *p* = 0.028; *β* = 0.151) In other words, in exchange for increasing a self-regulatory unit, the academic enthusiasm of English students increases by 0.151 units.

*H3*: Academic hope significantly affects English students’ academic enthusiasm.

The results of path analysis in Figure 2 and Table 5 show that the standard coefficient between academic hope and academic enthusiasm of English language learners is 0.197 and, according to the absolute value of *t*-test statistics which is equal to 3.289 and greater than 1.96, can be concluded with 99% probability that academic hope has a positive and significant effect on academic enthusiasm of English language learners (value of *p* = *0.001*; *β* = 0.197) In other words, in exchange for an increase in one unit of academic hope, English students’ academic enthusiasm increases by 0.197 units.

*H4*: Academic engagement significantly affects the academic enthusiasm of English language learners.

The results of path analysis in Figure 2 and Table 5 show that the standard coefficient between academic engagement and academic enthusiasm of English language learners is 0.208 and, according to the absolute value of t-test statistics which is equal to 4.186 and greater than 1.96, can be concluded with a 99% probability that academic engagement has a positive and significant effect on academic enthusiasm of language learners (value of *p* = 0.001; *β* = 0.208) In other words, in exchange for an increase in one unit of academic engagement, English students’ academic enthusiasm increases by 0.208 units.

*H5*: Teacher-student interaction significantly affects English students’ academic enthusiasm with the mediating role of self-regulation, academic hope, and engagement.

The results of the bootstrap test in Figure 2 and Table 5 show that the standard coefficient between teacher-student interaction and academic enthusiasm with the mediating role of self-regulation, academic hope, and academic engagement is 0.455. According to the level of significance, it can be concluded that with a 95% probability, teacher-student interaction having a positive and meaningful effect on academic enthusiasm with the mediating role of self-regulation, academic hope, and academic engagement (value of *p* = 0.013; *β* = 0.455). In other words, in exchange for increasing one unit of teacher-student interaction, academic enthusiasm with the mediating role of self-regulation, academic hope, and academic engagement increases by 0.455 units.

## Discussion

This research aimed to investigate the relationship between teacher-student interaction and academic enthusiasm with the mediation of academic self-regulation, academic engagement, and academic hope within the framework of a causal model using structural equation modeling. In general, the values of the fitness indices showed that the proposed model in this method has a good fit and can predict the changes in academic enthusiasm.

The results of the present study showed that teacher-student interaction is related to academic enthusiasm both directly and indirectly through academic hope and academic self-regulation. This finding is with the results of (Ibrahim et al., 2022; Jung and Ryu, 2022; Samadieh and Sadri, 2022) are aligned. In their research, Martin and Marsh (2006) concluded that academic enthusiasm has an inverse relationship with anxiety and a direct relationship with teacher-student interaction, self-regulation, and academic engagement. Also, the results of Grohman and Snyder’s (2022) research showed that teacher-student interaction is an indirect predictor of students’ academic enthusiasm. Rashidi et al. (2021) also showed in research that the quality of interaction between teachers and students plays a key role in students’ academic performance and a high level of teacher support leads to more academic interest, involvement, self-regulation, enthusiasm, and academic hope of students. In explaining this finding, it can be said that teachers play an essential role in the lives of children and teenagers. A strong and supportive interaction between teachers and students is the basis for the healthy growth of all students in school. Student-teacher interaction is the basis of successful adaptation to the social and academic environment and one of the important facilitative factors regarding academic difficulties and obstacles. When facing problems, students who have a good and constructive interaction with their teacher have more ability to solve and endure problems, which increases their hope, inner motivation, self-confidence, self-regulation, and academic enthusiasm.

Also, according to the research findings, academic hope is directly related to academic enthusiasm. This finding is with the results by Chena et al. (2020), Farid and Ashrafzade (2021), Snyder et al. (2002), and Wu and He (2022) are consistent. Sohrabi et al. (2021) concluded in their research that there is a positive and significant relationship between students’ hope and academic enthusiasm, and hopeful students have more academic enthusiasm. Also, the research results of Zhong et al. (2021) showed that people with higher academic enthusiasm have higher academic motivation and feel higher academic self-regulation and hope. Based on this, it can be concluded that hope seeks to achieve this goal by helping students to identify clear goals, categorize and create multiple paths to achieve these goals, and motivate them. Hopeful students use different ways to reach their goals as a worker and seem to be more problem-focused and have less avoidant coping than hopeless ones. Hopeful students have the stronger motivation and more energy to pursue their goals, and having a goal in life, believing in its realization, and trying to achieve the goal, increases the academic enthusiasm of students.

On the other hand, the research results showed that academic self-regulation is directly related to academic enthusiasm. This finding is with the results of the research of Teng and Zhang (2022) and Morosanova et al. (2016) are consistent. Fareley and Kimspoon (2014) concluded in their research that the growth of self-regulation reduces negative emotions and increases academic enthusiasm, and as a result, increases academic motivation and enthusiasm. Also, the results of Yun et al.’s research (2018) showed that academic self-regulation beliefs have a positive and significant relationship with academic enthusiasm. In explaining this finding, it can be said that self-regulation determines how people deal with obstacles and unpleasant experiences and the amount of effort and persistence they have to overcome obstacles. A sense of self-regulation motivates learners to engage with assignments, existing adverse conditions, and upcoming challenges. Students with high academic self-regulation beliefs perceive academic challenges less as insurmountable obstacles and are more motivated to remove obstacles and overcome the challenges of academic life. These students feel less depression and anxiety in traumatic and challenging situations and show more effort and persistence in overcoming challenges and solving problems, which reduces the probability of failure and the probability of academic success and enthusiasm increase.

The coefficients of the effects of academic engagement on academic enthusiasm were significant. The results of this study were aligned with the research of Ng et al. (2022) and Valadez et al. (2021). Academic enthusiasm leads to effective class and school activities participation, greater harmony with the school culture, and a good relationship with teachers and other students. This causes students to have more conflicts with academic subjects and school assignments, and as a result, their academic conflicts increase. Students who use more cognitive and metacognitive strategies show more stability in assignments and learning; this continuity in their assignments leads them to more success and creates a feeling of more success, enthusiasm, and motivation in them. The use of cognitive skills such as mental review, note-taking, summarizing, problem-solving, etc., increases the ability and learning power of the learners, and also causes a better application of the material. As a result, more motivation and enthusiasm to study is created in the students; therefore, cognitive strategies through the desire and enthusiasm to learn and have more effects on academic engagement.

In addition, the research results showed that student-teacher interaction is directly related to academic hope. This finding is consistent with the results of Roorda et al. (2017), Crouch et al. (2018), and Lan and Moscardino (2019) concluded in their research that teacher-student interaction causes satisfaction, participation in the class, academic engagement, hope, and more presence in the class. Also, the results of Crouch et al.’s research (Crouch et al., 2018) showed that students who establish supportive and friendly relationships with teachers have positive academic attitudes, satisfaction with school, academic engagement, and more hope to continue their education. Based on this, it can be concluded that the relationship with the teacher and the quality of acceptance by him improves the student’s motivation in academic activities as well as his emotional and social performance and is one of the important facilitating factors regarding difficulties. If the students receive and perceive the support from the teacher as one of the support resources available in the school, they are more able to bear and overcome the problems. Students who have warm and intimate interactions with their teachers have higher self-confidence, motivation, enthusiasm, and academic hope and have better academic performance than other students.

Also, the research results showed that teacher-student interaction is directly related to academic self-regulation. This finding is with the results of the research of Pintrich and de Groot (1990), Roorda et al. (2017), and Zimmerman and Schunk (2004) are consistent. In their research, Blasiman et al. (2017) concluded that there is a positive and significant relationship between self-regulation beliefs and teacher-student interaction. Also, the results of Scales et al.’s research (2019) showed that students with positive and constructive interaction with teachers have higher academic interests, academic involvement, success, self-regulation, and academic enthusiasm than other students. In explaining this finding, it can be said that in educational environments where students feel independent and belong, they are highly motivated and feel efficient and effective, but when the environment is controlled and the person does not feel that he is connected with others. His self-regulation decreases. He is not involved in doing things and is not motivated. The effectiveness of teaching is influenced by positive teacher-student communication more than it is caused by the cognitive teaching method, and a strong and supportive relationship between teacher and student is the basis for the healthy growth of all learners. In other words, with the teacher’s support for the students, they feel efficient and sufficient in their ability to learn lessons, and they attach importance to their lessons and see them as useful in their lives.

One of the important findings of the research was that the variables of academic self-regulation and academic hope play an important role in the relationship between student-teacher interaction and students’ academic enthusiasm. The results of some studies (Zimmerman, 2000; Scales et al., 2019; Roorda et al., 2019; Farid and Ashrafzade, 2021) show the existence of a relationship between student-teacher interaction with academic self-regulation and academic hope on the one hand, and the existence of a relationship between academic self-regulation and academic hope with enthusiasm. Therefore, assuming the mediating role of academic self-regulation and academic hope between student-teacher interaction and academic enthusiasm, which is the most important finding of the current research, is in some way consistent with the records of the current research. In general, it can be said that in this research, a model was presented for predicting academic enthusiasm based on teacher-student interaction, academic self-regulation, and academic hope. The results of the fit indices for this model showed that the model presented was a proportional fit with the research data has it. It can be concluded that with the improvement and development of teacher-student interaction, the level of academic self-regulation and academic hope of students increases, and all these factors together increase the academic enthusiasm of students.

## Conclusion

The purpose of the current study was to determine the causal explanation of academic enthusiasm based on the interaction of teachers and English language learners: self-regulation, academic hope, and academic engagement among English language learners. Our findings suggest that: The interaction between the teacher and the students has a significant effect on the academic enthusiasm of the English language students; self-regulation significantly affects the academic enthusiasm of English language learners.; academic hope significantly affects English language learners’ academic enthusiasm; academic engagement significantly affects English language learners’ academic enthusiasm; the interaction between the teacher and the students significantly affects the academic enthusiasm of the English language learners with the mediating role of self-regulation, academic hope, and academic engagement.

### Limitations, implications, and suggestions

Despite the important results of the present study, due to the limitations of this study, caution should be exercised in generalizing the results. Among the limitations of the current research, it can be mentioned that the statistical population of the research is limited to intermediate students, the lack of proof of the cause and effect relationship in the structural equation method, and the lack of confidence in the answers of the participants due to the self-report nature of the research tools. It is suggested that in future research, the role of other social and individual factors effective in academic enthusiasm should be investigated to test the assumptions raised in related theories, identify each factor’s contribution and examine the consequences of academic enthusiasm in the life of knowledge. Students and other groups of society should be addressed from different angles. Also, to test similar and generalized patterns, other samples with different ages and educational levels should be used. Also, considering the effect of teacher-student interaction on academic enthusiasm, it is suggested to hold effective communication skills training workshops for teachers and students during the academic year. On the other hand, considering the effect of academic hope and academic self-regulation on students’ academic enthusiasm, it is suggested that the educational system consider programs to improve the level of students’ academic hope, self-regulation, and academic engagement.

## Data availability statement

The raw data supporting the conclusions of this article will be made available by the authors, without undue reservation.

## Author contributions

All authors listed have made a substantial, direct, and intellectual contribution to the work and approved it for publication.

## Conflict of interest

The authors declare that the research was conducted in the absence of any commercial or financial relationships that could be construed as a potential conflict of interest.

## Publisher’s note

All claims expressed in this article are solely those of the authors and do not necessarily represent those of their affiliated organizations, or those of the publisher, the editors and the reviewers. Any product that may be evaluated in this article, or claim that may be made by its manufacturer, is not guaranteed or endorsed by the publisher.
